# How Early Is Early? Defining Functional Rehabilitation After Acute Achilles Tendon Rupture: A Narrative Review

**DOI:** 10.3390/jcm15176597

**Published:** 2026-08-26

**Authors:** Masanori Hashimoto, Youichi Yasui, Tomoyuki Nakasa, Hirotaka Kawano, Wataru Miyamoto

**Affiliations:** 1 Department of Orthopaedic Surgery, School of Medicine, Teikyo University, 2-11-1 Kaga, Itabashi-ku, Tokyo 173-8605, Japanmiyamoto@med.teikyo-u.ac.jp (W.M.); 2 Department of Musculoskeletal Reconstruction and Biomaterials, Graduate School of Biomedical and Health Sciences, Hiroshima University, 1-2-3 Kasumi, Minami-ku, Hiroshima 734-8551, Japan

**Keywords:** Achilles tendon rupture, functional rehabilitation, early weightbearing, nonoperative treatment, rehabilitation timing, tendon healing

## Abstract

Early functional rehabilitation after acute Achilles tendon rupture is increasingly used in nonoperative protocols, but the definition of “early” remains highly heterogeneous across the literature and no standardized timing threshold has been established. This narrative review clarifies the conceptual and operational definitions of early rehabilitation and examines the unresolved timing-threshold problem. Existing evidence supports early functional rehabilitation without a demonstrated increase in rerupture risk compared with delayed immobilization. Early rehabilitation may improve short-term functional recovery; however, clear long-term superiority over more delayed strategies has not been demonstrated. Substantial heterogeneity persists in the initiation of weightbearing and range of motion and in orthosis duration. Although weightbearing within four weeks is commonly used as an operational definition of early rehabilitation for evidence synthesis, it is not a biologically or clinically validated threshold. The literature also lacks granular evidence defining a timing–response relationship or an optimal initiation window. Current evidence therefore supports early functional rehabilitation as a reasonable strategy, but future research should identify validated timing–response relationships and patient-specific strategies.

## 1. Introduction

Acute Achilles tendon rupture is a common orthopaedic injury, and nonoperative treatment with functional rehabilitation has become an increasingly accepted management strategy [1,2]. Accelerated protocols and early weightbearing are now widely used in clinical practice [3,4], and evidence from randomized trials and secondary syntheses supports early functional rehabilitation without a demonstrated increase in rerupture risk [5,6,7,8,9]. Some studies report earlier improvements in function or quality of life, whereas clear long-term superiority over more delayed strategies has not been consistently demonstrated [6,8,9]. Protocols nevertheless remain heterogeneous in the timing and progression of weightbearing and ankle motion and in orthosis use [6,9,10]. It is difficult to evaluate the effect of timing alone using traditional methods that simply compare “early” and “delayed” groups, because rehabilitation timing is embedded within a multicomponent rehabilitation protocol in which factors such as weightbearing, ankle range of motion, orthosis use, and ankle position often change simultaneously. Therefore, a narrative approach is suitable to review how past studies defined timing, consider these interrelated protocol components, and evaluate whether current evidence supports a clinically meaningful threshold for timing.

Despite this acceptance, the concept of “early” rehabilitation lacks a standardized definition. Many studies rely on operational thresholds, such as weightbearing within four weeks of treatment, but this boundary has not been biologically or clinically validated [6,7]. Although rehabilitation timing has been examined in several trials, most studies compare only discrete early and delayed strategies, and several rehabilitation components are frequently altered simultaneously. Thus, the unresolved problem is not an absence of timing research, but the lack of an established granular timing–response relationship from which an optimal initiation window could be derived. Consequently, it remains unclear whether earlier initiation yields superior clinical outcomes. More fundamentally, the commonly used four-week boundary should be interpreted as an operational definition used to categorize heterogeneous protocols rather than as a biologically or clinically validated threshold [6,9,10].

Accordingly, this narrative review examines how “early” rehabilitation has been defined, evaluates the evidentiary basis of the four-week boundary, distinguishes direct nonoperative evidence from indirect mechanistic evidence derived from surgically repaired tendons, and identifies priorities for future research.

## 2. Literature Search and Review Approach

This article is a narrative review rather than a systematic review. A targeted PubMed literature search was conducted on 11 August 2026, with a primary focus on rehabilitation timing after nonoperative treatment of acute Achilles tendon rupture.

The following PubMed search strategies were used (abbreviations: RCT, randomized controlled trial; MRI, magnetic resonance imaging):

Core nonoperative rehabilitation search: (“Achilles Tendon”[Mesh] OR “Achilles tendon”[tiab]) AND rupture*[tiab] AND (nonoperativ*[tiab] OR non-operativ*[tiab] OR nonsurgical[tiab] OR non-surgical[tiab] OR conservative[tiab]) AND (rehabilitat*[tiab] OR “functional rehabilitation”[tiab] OR weightbearing[tiab] OR “weight bearing”[tiab] OR mobilization[tiab] OR mobilisation[tiab] OR “ankle motion”[tiab])

Timing-focused search: (“Achilles tendon”[tiab] AND rupture*[tiab]) AND (early[tiab] OR immediate[tiab] OR delayed[tiab] OR late[tiab] OR timing[tiab]) AND (weightbearing[tiab] OR “weight bearing”[tiab] OR “ankle motion”[tiab] OR mobilization[tiab] OR mobilisation[tiab] OR rehabilitation[tiab]) AND (nonoperativ*[tiab] OR non-operativ*[tiab] OR nonsurgical[tiab] OR conservative[tiab])

RCT-focused search: (“Achilles tendon”[tiab] AND rupture*[tiab]) AND (early[tiab] OR immediate[tiab] OR delayed[tiab] OR timing[tiab]) AND (weightbearing[tiab] OR “weight bearing”[tiab] OR “ankle motion”[tiab] OR rehabilitation[tiab]) AND (randomized controlled trial[pt] OR randomized[tiab] OR randomised[tiab])

Four-week threshold search: (“Achilles tendon”[tiab] AND rupture*[tiab]) AND (“4 weeks”[tiab] OR “four weeks”[tiab] OR threshold[tiab] OR cut-point[tiab] OR timing[tiab]) AND (weightbearing[tiab] OR rehabilitation[tiab] OR “ankle motion”[tiab])

Imaging search: (“Achilles tendon”[tiab] AND rupture*[tiab]) AND (ultrasound[tiab] OR ultrasonography[tiab] OR MRI[tiab] OR “magnetic resonance”[tiab]) AND (gap[tiab] OR apposition[tiab] OR position[tiab] OR dynamic[tiab] OR healing[tiab] OR mechanical[tiab] OR rehabilitation[tiab])

Key studies were additionally verified using author- and title-based PubMed searches. Particular emphasis was placed on randomized controlled trials, followed by systematic reviews/meta-analyses and clinically relevant cohort studies. The direct clinical synthesis focused on studies of acute Achilles tendon rupture managed nonoperatively that evaluated rehabilitation timing or major rehabilitation components, including weightbearing, ankle motion, orthosis use, and ankle position. Studies of surgically repaired tendons were used only as indirect mechanistic evidence when discussing tendon elongation, loading, or healing biology; they were not treated as direct evidence for nonoperative rehabilitation timing.

Because this narrative review used a targeted literature search rather than a formal systematic-review methodology, Preferred Reporting Items for Systematic Reviews and Meta-Analyses (PRISMA)-based screening, study-selection counts, protocol registration, and formal risk-of-bias assessment were not performed.

Generative artificial intelligence (AI) tools, including ChatGPT (GPT-5.6 Sol, OpenAI), Gemini (Gemini 3.5 Flash, Google), and Claude (Claude Sonnet 5, Anthropic), were used during manuscript preparation for language refinement, structural editing, and critical review support. The PubMed searches reported above, verification of cited references, and all final scientific interpretation and judgment were performed by the authors. These tools were not used to generate original data, perform statistical analyses, independently determine study eligibility, or make scientific conclusions.

## 3. Historical Evolution of Functional Rehabilitation

The nonoperative management of acute Achilles tendon rupture has changed substantially over the past several decades [2,11]. Traditional conservative treatment was based on prolonged immobilization of the ruptured tendon. This approach usually involved rigid plaster casting with the ankle positioned in equinus. Weightbearing was commonly delayed, and immobilization periods of approximately six to eight weeks were widely used [1,2]. Early ankle motion was generally avoided because the healing tendon was considered mechanically vulnerable during the early reparative phase [12].

This traditional model had a clear protective rationale. Immobilization was intended to reduce tension across the rupture site. It was also intended to minimize tendon gapping, tendon elongation, and rerupture during early healing [2,13]. However, prolonged casting had important clinical disadvantages. Prolonged immobilization and non-weightbearing can limit mobility and increase reliance on assistive devices. They may also contribute to clinical disadvantages such as ankle stiffness, calf muscle atrophy, venous thromboembolic risk, and delayed return to daily activities. These limitations encouraged clinicians to seek treatment strategies that could protect the tendon while allowing earlier functional recovery [2,3].

Functional rehabilitation emerged as a response to these limitations [2,3,8]. The key conceptual shift was not simply that patients were allowed to walk earlier. Rather, functional rehabilitation introduced the idea that controlled loading could be compatible with tendon healing when the ankle was protected in plantar flexion [10]. This approach attempted to balance two clinical goals. The first goal was mechanical protection of the healing tendon. The second goal was preservation of limb function through earlier mobility and progressive loading [2,3,8,10,14].

Functional braces represented an important step in this transition [2,3]. Compared with rigid plaster casts, braces provided a more adaptable treatment environment. They could maintain the ankle in a protected position while permitting some degree of functional use. This helped move nonoperative care away from absolute immobilization. It also introduced the practical concept that tendon protection and functional rehabilitation were not mutually exclusive [2,8,10,13].

Walking boots further changed the delivery of conservative treatment [2,3]. Removable boots allowed staged adjustment of ankle position during the healing process. Heel wedges could be reduced gradually as treatment progressed. This enabled a controlled transition from equinus toward a more neutral ankle position. Walking boots also made protected weightbearing easier to implement in daily practice. They improved patient convenience and allowed rehabilitation protocols to become more structured and reproducible [2,3,11,15].

Early weightbearing then became a major component of accelerated rehabilitation. However, early weightbearing was not applied uniformly across studies [1,2,3,6,9]. Some protocols permitted immediate weightbearing as tolerated from the first day of treatment [1,2,3,13]. Other protocols used progressive partial weightbearing before full loading was allowed [2,3,14]. These differences are clinically important. The term “early weightbearing” often referred to one part of a broader treatment package. This package could also include a specific brace or boot, a heel-wedge reduction schedule, a defined orthosis duration, and variable timing of ankle range-of-motion exercises [2,3,14].

Large randomized trials and pragmatic treatment protocols helped support this transition from rigid casting to functional bracing and walking-boot-based care [2,3,4]. These studies showed that selected accelerated protocols can be used without a demonstrated increase in rerupture risk compared with more traditional immobilization strategies [1,2,3,6,9]. Some trials also suggested early benefits in mobility, quality of life, or short-term functional recovery [2,3]. These findings helped normalize early functional treatment in clinical practice.

This historical evolution is important for interpreting the modern literature. The term “early rehabilitation” originally developed as a clinical contrast to prolonged immobilization and delayed weightbearing. It did not arise from evidence identifying a precise biological threshold for safe or optimal loading. As a result, contemporary studies often use “early” to describe heterogeneous combinations of functional bracing, walking boots, controlled ankle motion, and protected weightbearing. The historical shift from six- to eight-week casting toward functional rehabilitation therefore explains both the clinical adoption of early protocols and the persistent uncertainty regarding the exact meaning of “early” [2,3,6,7,9].

## 4. What Is “Early” Rehabilitation?

The concept of “early” rehabilitation historically emerged as a clinical contrast to prolonged immobilization and non-weightbearing [1,2]. Zellers et al. documented substantial heterogeneity in how the term has been used across the literature [16]. Within this literature, rehabilitation timing has been studied through discrete comparisons. Barfod et al. compared full weightbearing from day 1 with non-weightbearing for 6 weeks [1]; Young et al. compared weightbearing with non-weightbearing casts [17]; Korkmaz et al. compared partial weightbearing from the day of treatment with 4 weeks of non-weightbearing [18]; and Barfod et al. compared controlled ankle motion beginning around day 14 with immobilization [5]. These studies demonstrate that rehabilitation timing is not an unstudied variable; however, they do not provide a multi-level or continuous timing–response analysis across closely spaced initiation points. For terminological clarity, ‘immediate’ rehabilitation here denotes loading or motion begun on day 0–1; ‘early’ rehabilitation denotes initiation within the broader early window used in systematic reviews; and ‘accelerated’ rehabilitation refers to a protocol that advances one or more rehabilitation components relative to traditional prolonged immobilization. These terms are operational descriptors rather than validated biological thresholds.

Because “early functional rehabilitation” serves as an umbrella concept, it encompasses multiple distinct therapeutic components [8,9,16]. The literature reveals substantial differences in how these components are applied. Weightbearing strategies range from immediate full loading on day one to progressive, phased weightbearing protocols [2,3,4]. Similarly, the initiation of range of motion varies; some protocols utilize functional braces that permit immediate, unprescribed ankle motion, while others prescribe specific controlled motion exercises beginning at week three [3,5]. The orthosis type itself adds another variable, contrasting flexible functional braces or walking boots with rigid plaster casts [2,3]. The timing of exercise or strengthening also varies across rehabilitation protocols [16], while return-to-activity milestones are not uniformly defined or reported across studies [6,9].

The overlapping nature of these distinct components explains why definitions of “early” rehabilitation remain highly heterogeneous across the literature [6,9,10]. Clinical trials frequently combine different weightbearing timelines, range-of-motion initiation points, and orthosis types into complex, multimodal packages [2,3,5]. Therefore, when systematic reviews attempt to compare “early” versus “delayed” treatments, they are often comparing highly variable combinations of mechanical stimuli rather than isolating a single timing variable [6,9].

Systematic reviews have used different working definitions of “early.” Zellers et al. reported that the median timing of weightbearing and exercise onset was 2 weeks (interquartile range [IQR], 0–3 weeks) across all included studies, including both surgical and nonsurgical management. In the nonsurgical subgroup, weightbearing onset occurred at a median of 0 weeks (IQR, 0–2 weeks), whereas exercise onset occurred at a median of 3 weeks (IQR, 2–4 weeks) [16]. Ghaddaf et al. defined early weightbearing as initiation within 4 weeks for meta-analytic eligibility [6]. This cut-point facilitates evidence synthesis but was not prospectively validated as an optimal biological or clinical threshold.

Such protocol boundaries are useful for classifying heterogeneous studies, but they should not be conflated with physiological milestones. In individual trials, timing is also linked to orthosis design, ankle position, heel-wedge progression, and permitted motion, which limits causal attribution to timing alone [2,3,5,10].

A critical distinction must therefore be made between an operational definition and a biologically meaningful timing threshold. An operational definition groups variable protocols for comparison or evidence synthesis. A biologically meaningful threshold would require evidence that a particular initiation window consistently changes healing or clinical outcomes while the remaining rehabilitation components are held sufficiently constant. Such evidence has not yet been established.

## 5. Biological Rationale and Its Limitations

The transition toward early functional rehabilitation is influenced by the fundamental principles of tendon biology. Tendon healing is generally described as progressing through overlapping inflammatory, proliferative, and remodeling phases [19,20,21]. Experimental and mechanobiological literature suggests that controlled loading can influence collagen synthesis and tissue organization [22,23]. These phases overlap rather than occurring at sharply defined clinical time points, so basic tendon biology does not identify a single calendar threshold for loading.

Direct nonoperative mechanistic evidence also suggests that the biological response to early loading is more complex than a simple “earlier is better” model. In a randomized trial of 40 nonsurgically treated ruptures, Rendek et al. evaluated additional graded tensile loading beginning approximately 2 weeks after injury. The intervention altered tendon morphology and mechanical properties without demonstrating a simple improvement in tendon stiffness, suggesting that the structural response to additional early loading is complex [24].

Concerns about tendon elongation remain important, but much of the detailed elongation and loading literature comes from surgically repaired tendons [25,26,27,28,29,30,31,32]. Surgical trials and cohorts have examined accelerated versus delayed loading, tendon lengthening, muscle atrophy, and long-term strength [27,28,29,30,31,32]. These studies are useful as indirect mechanistic evidence, but the mechanical environment after surgical repair differs from that of nonoperative treatment; their findings should therefore not be interpreted as direct evidence for an optimal nonoperative initiation time.

Imaging can characterize tendon apposition and healing morphology but does not currently provide a validated readiness threshold for advancing rehabilitation. Dynamic ultrasonography has shown that the measured rupture gap changes substantially with ankle and knee position, indicating that a single static gap measurement is position dependent [33]. Dynamic ultrasound has also been used as a treatment-selection tool, but this does not constitute validation of a cut-point for progressing weightbearing or motion [34]. Longitudinal ultrasonographic and MRI assessment likewise has not established imaging appearance as a surrogate for mechanical readiness [35]. No prospectively validated ultrasound-gap or MRI-signal threshold for advancing rehabilitation was identified in the targeted literature search. Taken together, the biological and imaging literature supports careful, protected loading but does not define an optimal calendar start point.

## 6. Does Earlier Mean Better?

Current evidence suggests that early functional rehabilitation may provide transient early functional or quality-of-life benefits. The UK Study of Tendo Achilles Rehabilitation (UKSTAR) trial reported better early patient-reported function with functional bracing, and Maempel et al. reported several early advantages with a walking-boot strategy; however, clear superiority was not demonstrated at later follow-up [2,3]. Meta-analytic evidence is similarly mixed, with some early functional advantages but no consistent long-term superiority [6,8,9].

Direct nonoperative studies of weightbearing or motion timing also generally fail to demonstrate sustained superiority of the earlier strategy. Barfod et al. found no statistically significant improvement in 1-year Achilles Tendon Total Rupture Score (ATRS) with immediate weightbearing [1]. Young et al. randomized patients to weightbearing or non-weightbearing casts, providing another direct comparison of loading strategies without defining an optimal initiation time [17]. Korkmaz et al. reported no significant between-group differences in functional scores or rerupture at 12 months when same-day partial weightbearing was compared with 4 weeks of non-weightbearing [18]. Early controlled motion likewise did not demonstrate superior 1-year outcomes in Barfod et al. [5]. Taken together, these studies support the feasibility of earlier rehabilitation but do not establish a monotonic timing–response relationship in which earlier initiation consistently produces better outcomes. Key evidence regarding rehabilitation-timing comparisons is summarized in Table 1.

## 7. The Unresolved Timing–Response Relationship

Current evidence supports early functional rehabilitation as a reasonable nonoperative strategy without demonstrating consistent superiority of the earliest initiation strategy [1,2,3,5,6,9,17,18]. The central unresolved issue is therefore not whether rehabilitation timing has been studied, but whether existing comparisons can define a clinically meaningful timing–response relationship.

The main limitation is the structure of the comparisons. Existing studies generally contrast two discrete protocols—for example, immediate or same-day weightbearing versus several weeks of non-weightbearing, or controlled motion beginning at one prespecified time point versus immobilization [1,5,17,18]. These designs are clinically useful, but they cannot determine whether outcomes change progressively across closely spaced initiation times.

Moreover, timing is frequently bundled with differences in orthosis, ankle position, permitted motion, and immobilization duration [2,3,10]. Consequently, any observed effect may reflect the rehabilitation package rather than initiation timing alone.

A granular timing–response relationship has therefore not been established. What is missing is not timing research itself, but trials or high-quality prospective studies comparing closely spaced initiation windows while keeping the remaining rehabilitation protocol as similar as possible.

Accordingly, review-defined and protocol-specific temporal boundaries should be regarded as operational constructs unless and until they are prospectively validated as biologically or clinically meaningful cut-points.

Current evidence thus supports early functional rehabilitation as a practical option but leaves the optimal initiation window unresolved. This distinction allows the literature to support early rehabilitation without implying that the earliest possible start is necessarily best.

## 8. Clinical Implications

The current body of evidence supports early functional rehabilitation as a reasonable approach for the nonoperative management of acute Achilles tendon rupture, without a demonstrated increase in rerupture in the major comparative studies [1,2,3,5,6,9,17,18]. For many patients, prolonged strict non-weightbearing immobilization is therefore not the only acceptable strategy. Clinicians may use accelerated or immediate-weightbearing protocols when appropriate within a structured treatment pathway, while recognizing that the evidence supporting their feasibility and safety is stronger than the evidence for long-term superiority.

This overall safety evidence must be interpreted with clinical nuance. Clinicians should avoid the assumption that “early” automatically means “as early as possible.” Earlier loading or motion has not demonstrated consistent long-term superiority over more delayed strategies [1,2,3,5,6,9,17,18].

Because current evidence does not establish a validated patient-specific timing or progression algorithm, decisions regarding rehabilitation progression may need to incorporate pragmatic clinical considerations in addition to protocol timing. Factors such as pain, swelling, orthosis fit, safe gait, adherence, baseline activity level, rupture characteristics, and access to follow-up may reasonably be considered in practice. These factors should be viewed as pragmatic clinical considerations rather than validated criteria for determining a specific initiation time.

In practice, patient- and context-related factors may influence the feasibility of applying an early functional protocol. A relatively healthy patient who can understand the rehabilitation plan, use a walking boot correctly, and attend follow-up visits is different from an older patient with impaired balance, cognitive decline, neuropathy, frailty, or limited home support [36]. In elderly or low-activity patients, the expected benefit of faster early mobility must be balanced against fall risk, difficulty managing heel wedges, and the possibility of poor adherence. In such circumstances, clinicians may pragmatically consider a more cautious progression, although this approach has not been validated as a patient-specific timing rule [2,3,37,38]. Conversely, younger athletic patients may prioritize earlier gait recovery, maintenance of conditioning, and return to work or sport. However, they should also be counseled that an accelerated protocol does not necessarily guarantee superior one-year function, calf strength, or return-to-sport outcomes [3,7,8].

Several practical problems also determine whether early rehabilitation is clinically feasible. Functional braces and walking boots can facilitate protected loading, but only if they fit well and are used consistently. Poor orthosis fit may cause heel irritation, pressure injury, skin maceration, or pain, especially in older patients or those with diabetes, peripheral vascular disease, or sensory disturbance [39,40]. Pain during loading should not be interpreted simply as a reason to abandon rehabilitation, but it should prompt reassessment of boot position, wedge height, gait pattern, swelling control, and patient understanding of restrictions. Compliance is equally important. A protocol that is safe in a clinical trial may become unsafe if the patient removes the boot frequently, bears weight without protection, or advances ankle motion beyond the prescribed range [2,13].

Venous thromboembolic risk is another important clinical consideration. Prolonged immobilization and non-weightbearing can reduce calf muscle pump activity and increase dependence on assistive devices. Early protected weightbearing may improve mobility and may theoretically reduce immobility-related complications, but it should not replace formal risk assessment [2,10,41]. Patients with previous venous thromboembolism, malignancy, marked obesity, thrombophilia, prolonged travel, or very limited mobility may require individualized thromboprophylaxis decisions according to local practice [41,42]. Thus, early rehabilitation should be viewed as one component of comprehensive medical management rather than as a stand-alone solution.

Although no validated readiness criteria are currently available, pragmatic considerations when applying early protected weightbearing within an established protocol may include whether the ankle can be maintained in an appropriate protected position using a boot or brace, whether pain and swelling are manageable, whether the patient can mobilize safely with or without assistive devices, and whether reliable follow-up is available [2,11,13]. Imaging can characterize tendon apposition and healing morphology in selected cases, but no validated imaging cut-point currently defines mechanical readiness to advance weightbearing or ankle motion [33,34,35]. Patient education should emphasize that current evidence supports convenience, mobility, and potential short-term recovery benefits, whereas clear long-term superiority of earlier initiation has not been demonstrated.

Finally, these clinical realities should inform shared decision-making. Patients can be told that early functional protocols may improve convenience and some early recovery measures, while current evidence does not demonstrate consistent long-term superiority of immediate or accelerated initiation over more delayed approaches [1,2,3,5,6,8,9,17,18].

## 9. Future Directions: Toward a Practical Clinical Framework

Several research priorities follow from the preceding analysis. The most fundamental need is for timing–response studies that treat rehabilitation initiation as a graded exposure rather than a simple early-versus-delayed label [6,9,16]. Trials comparing closely spaced initiation windows—for example, week 1, week 2, and week 3—while holding orthosis, ankle position, permitted motion, and loading progression as constant as possible would help determine whether a clinically meaningful timing gradient exists. Until such data are available, a single initiation point should be regarded as a pragmatic protocol choice rather than a validated biological threshold.

A second priority is the objective assessment of tendon elongation and mechanical recovery during healing. Standardized measures of tendon length, gap behavior, resting ankle angle, and functional performance could clarify whether timing-related differences in tissue behavior translate into meaningful outcomes. Ultrasound and MRI may be useful candidate biomarkers, but their value should be tested prospectively because current imaging findings do not define validated progression thresholds [33,34,35].

A third priority is patient-specific rehabilitation. Current protocols apply broadly similar timelines to clinically heterogeneous patients [6,9]. Future studies could examine whether loading progression should differ for older adults, low-activity patients, athletes, and patients at high risk of complications. Elderly and low-activity patients may prioritize safety, adherence, and fall avoidance, whereas athletes may prioritize earlier gait recovery and return to conditioning [3,7]. Whether these differing priorities justify distinct timing strategies has not been formally tested, and any subgroup-specific recommendation therefore remains provisional.

Progress will also depend on methodological standardization. Heterogeneous outcome measures and follow-up intervals currently limit pooling across trials [6,9]. Consistent use of validated instruments, uniform assessment time points, and explicit reporting of each protocol component—weightbearing status, range-of-motion permission, orthosis type, and heel-wedge schedule—would allow future syntheses to isolate the effect of timing rather than the effect of an entire treatment package [2,3]. Adequately powered trials with prespecified timing subgroups would be needed to detect the modest differences that a timing–response relationship, if present, is likely to produce.

Pending such evidence, the specific initiation point should be individualized within a structured protocol and regarded as a pragmatic clinical decision rather than as a time point proven to optimize long-term function.

Finally, pragmatic trial designs and registry-based studies may help capture real-world variation in rehabilitation timing across larger populations. Combining such approaches with standardized measures of tendon elongation, mechanical recovery, and functional outcomes could provide the granular evidence that is currently lacking. The objective should not be to replace one operational threshold with another, but to determine whether rehabilitation timing can ultimately be linked to measurable healing characteristics and patient-specific factors rather than relying solely on fixed calendar-based boundaries.

## 10. Discussion and Perspectives

The shift from prolonged immobilization to early functional rehabilitation represents a major historical evolution in the nonoperative management of acute Achilles tendon ruptures [2,3,4]. However, this transition has created an important terminology issue. As demonstrated throughout this review, “early” rehabilitation currently functions as a broad operational umbrella rather than a precise biological concept [6,9,16].

The existing literature generally supports early weightbearing and functional rehabilitation without a demonstrated increase in rerupture and suggests that some protocols may provide transient benefits during early recovery [5,6,7,8,9]. Yet a critical distinction remains: demonstrating that an earlier strategy is feasible and not clearly more harmful does not establish that earlier initiation is inherently superior [1,2,3,5,17,18].

The central unresolved issue is therefore not whether timing has been studied, but what existing timing studies can establish. Discrete comparisons have evaluated immediate or day-1 weightbearing, same-day partial weightbearing, and controlled motion beginning at a prespecified early time point [1,5,17,18], while larger pragmatic trials have compared bundled functional-rehabilitation packages [2,3]. Because initiation timing, loading dose, ankle motion, orthosis design, and ankle-position progression are frequently linked, these studies have not established a granular timing–response relationship. Systematic reviews use working definitions to synthesize this heterogeneous evidence; the four-week boundary is one such operational definition rather than a prospectively validated biological or clinical threshold [6,16].

This review should also be interpreted in light of its methodological limitations. As a narrative review based on a targeted PubMed literature search, it was not designed as an exhaustive systematic review, and no formal risk-of-bias assessment was performed. Its purpose was instead to clarify the concept of rehabilitation timing, examine how timing is embedded within heterogeneous rehabilitation protocols, and identify the limitations of the current evidence regarding a clinically meaningful timing threshold.

**Figure 1:**
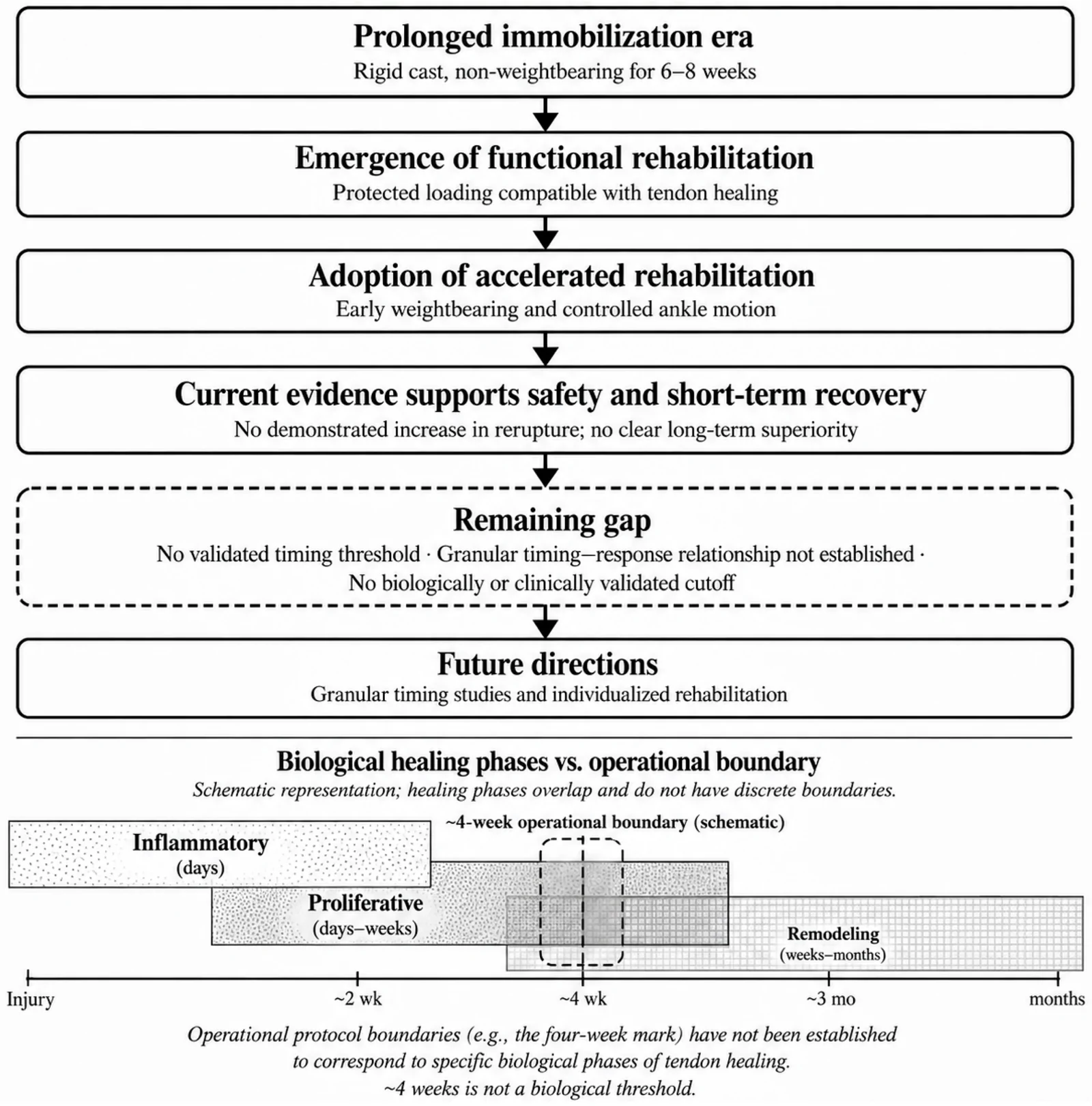
Conceptual framework contrasting the clinical adoption of early functional rehabilitation with the absence of a validated timing threshold after acute Achilles tendon rupture. The lower panel indicates that operational protocol boundaries, such as the four-week mark, have not been established to correspond to specific biological phases of tendon healing. Abbreviations: wk, weeks; mo, months.

Future research should move beyond broad binary comparisons and evaluate closely spaced timing windows, patient-specific rehabilitation strategies, tendon elongation, and candidate imaging or mechanical markers. The objective is not to replace one operational boundary with another, but to determine whether measurable healing characteristics can contribute to a reproducible timing–response model for nonoperative rehabilitation. A conceptual summary of these findings is provided in Figure 1.

## 11. Conclusions

Current evidence supports early functional rehabilitation as a reasonable strategy for nonoperatively treated acute Achilles tendon ruptures, without a demonstrated increase in rerupture risk; however, the concept of “early” remains operationally heterogeneous across the literature. Timing has been studied through discrete and often bundled comparisons, yet a granular timing–response relationship has not been established. Earlier weightbearing or motion may provide short-term benefits, while clear long-term superiority over more delayed strategies has not been demonstrated. Operational timing boundaries used in the literature should therefore not be interpreted as biologically or clinically validated thresholds. Future research should more precisely define timing–response relationships and evaluate patient-specific rehabilitation strategies.

## Figures and Tables

**Table 1:** Evidence hierarchy and rehabilitation-timing comparisons in key studies relevant to nonoperative acute Achilles tendon rupture.

Evidence Category	Study	Design/Population	Timing or Protocol Comparison	Main Finding/Relevance to Timing
Direct randomized nonoperative evidence	Barfod et al., 2014 [1]	Assessor-blinded RCT; 60 randomized; acute ATR, nonoperative	Full WB from day 1 vs. NWB for 6 weeks; both groups otherwise followed the same dynamic protocol with controlled early motion	No significant difference in 12-month ATRS (73 vs. 74; *p* = 0.81) or rerupture (3 vs. 2; *p* = 1.0). Direct binary weightbearing-timing comparison; no demonstrated 1-year superiority of immediate WB.
Direct randomized nonoperative evidence	Young et al., 2014 [17]	RCT; 84 randomized; acute ATR, nonoperative	Weight-bearing cast vs. non-weight-bearing cast for 8 weeks; follow-up to 2 years	At 2 years, reruptures were 1/37 vs. 2/37 (*p* = 0.62). WB was associated with less subjective stiffness at 1 year, but no significant differences were found in return to work, Leppilahti score, pain, satisfaction, or return to sport. Direct comparison; no optimal initiation threshold defined.
Direct randomized nonoperative evidence	Korkmaz et al., 2015 [18]	RCT; 47 randomized; acute ATR, nonoperative	Partial WB from the day of treatment (*n* = 23) vs. NWB for the first 4 weeks (*n* = 24); otherwise identical conservative treatment	No significant between-group differences in AOFAS, ATRS, or PAS at 6 or 12 months; reruptures were 4/23 vs. 3/24 (*p* = 0.81). Direct same-day vs. 4-week-delay comparison; does not validate a 4-week threshold.
Direct randomized nonoperative evidence	Barfod et al., 2020 [5]	Assessor-blinded RCT; 130 randomized; acute ATR, nonoperative	From day 14 (beginning of week 3), full WB was allowed in both groups; controlled ankle motion from week 3–8 vs. no ankle motion until week 9.	No demonstrated 1-year benefit of early controlled motion in ATRS, heel-rise work, tendon elongation, or rerupture (6 vs. 7). Direct motion-start comparison on a shared WB background.
Direct randomized nonoperative evidence	Costa et al., 2020 (UKSTAR) [2]	Pragmatic multicentre superiority RCT; 540 randomized at 39 UK hospitals; acute ATR, nonoperative	Functional brace permitting immediate WB vs. plaster-cast strategy; ankle/foot position progressed during 8 weeks of treatment	ATRS favored the functional brace at 8 weeks, but no significant difference was demonstrated at 9 months; reruptures were 13 vs. 17. Bundled brace/WB comparison rather than timing alone.
Direct randomized nonoperative evidence	Maempel et al., 2020 [3]	Single-centre nonblinded RCT; 140 randomized; acute ATR, nonoperative	Immediate full WB in a walking boot for 8 weeks vs. 10 weeks of cast-based treatment, with non-WB for the first 8 weeks followed by full WB in a neutral cast for 2 weeks.	Several PROMs favored the boot at 6 months; no significant differences in the reported PROMs were demonstrated at 1 year. Reruptures were 5 vs. 11 (*p* = 0.075). Bundled rehabilitation comparison rather than timing alone.
Observational nonoperative evidence	Aujla et al., 2019 (LAMP) [4]	Prospective cohort; 442 nonoperative patients; minimum 12-month follow-up	Eight-week VACOped functional dynamic protocol with immediate full WB	Nine reruptures occurred among 442 patients (2%); ATRS at 12 months or more was available in 234/442. Demonstrates feasibility of standardized immediate WB in clinical practice, without a randomized comparator.
Secondary evidence	Zellers et al., 2019 [16]	Systematic review; 174 studies, 9098 participants; mixed operative/nonoperative	Descriptive synthesis of WB and exercise/strengthening initiation across heterogeneous protocols	All included studies (surgical and nonsurgical): WB and exercise onset, median 2 weeks (IQR, 0–3 weeks). Nonsurgical subgroup: WB, 0 weeks (IQR, 0–2 weeks); exercise, 3 weeks (IQR, 2–4 weeks). Documents substantial heterogeneity in the definition/components of early functional rehabilitation, descriptive rather than causal evidence on timing.
Secondary evidence	Liu et al., 2021 [8]	Meta-analysis; 19 trials, 1758 participants; mixed operative/nonoperative	Early functional rehabilitation vs. traditional immobilization; protocols varied	No significant difference was demonstrated in rerupture, return to work, or sporting activity; an early ATRS benefit was reported, while clear longer-term superiority was not established. Mixed treatment modalities limit direct inference for nonoperative timing.
Secondary evidence	Dai et al., 2021 [9]	Systematic review/meta-analysis; 8 RCTs, 978 patients; nonoperative	Early WB with or without functional ankle motion vs. traditional immobilization/NWB	No significant differences were demonstrated in rerupture, return to sport/work, functional outcomes, or complications across the principal comparisons. Heterogeneous regimens do not identify a single optimal initiation time.
Secondary evidence	Jamjoom, 2021 [10]	Systematic review; 18 publications, 1068 patients; nonoperative	Component-based synthesis of early WB (≤4 weeks), controlled motion, and early orthosis removal (≤8 weeks)	Weightbearing, motion, and orthosis-removal timing were analyzed separately. Early orthosis removal (≤8 weeks) was associated with a higher pooled rerupture rate (9.6% vs. 2.7%), although heterogeneous protocols preclude defining a validated timing threshold.
Secondary evidence	Ghaddaf et al., 2022 [6]	Systematic review/meta-analysis; 9 RCTs, 1046 participants; conservative/nonoperative	Early WB defined as initiation within 4 weeks vs. late WB; heterogeneous protocols pooled	No significant differences were demonstrated in rerupture, ATRS, return to preinjury sport, time to return to work, or adverse events. The 4-week cut-point is a review-defined operational criterion, not a prospectively validated threshold.

Studies are grouped as direct randomized nonoperative evidence, observational nonoperative evidence, and secondary evidence. Mechanistic studies focused on tendon structural or biological responses, including Rendek et al. (2022) [24], and studies of surgically repaired tendons are discussed separately in the biological/mechanistic section and are not included in this table. Abbreviations: AOFAS, American Orthopaedic Foot & Ankle Society; ATR, Achilles tendon rupture; ATRS, Achilles Tendon Total Rupture Score; IQR, interquartile range; LAMP, Leicester Achilles Management Protocol; NWB, non-weightbearing; PAS, Physical Activity Scale; PROMs, patient-reported outcome measures; RCT, randomized controlled trial; UKSTAR, UK Study of Tendo Achilles Rehabilitation; WB, weightbearing.

## Data Availability

All data discussed in this review are available from the published articles cited in the reference list. No new datasets were generated for this study.
